# Re-Meandering of Lowland Streams: Will Disobeying the Laws of Geomorphology Have Ecological Consequences?

**DOI:** 10.1371/journal.pone.0108558

**Published:** 2014-09-29

**Authors:** Morten Lauge Pedersen, Klaus Kevin Kristensen, Nikolai Friberg

**Affiliations:** 1 Department of Civil Engineering, Aalborg University, Aalborg, Denmark; 2 Ringkjøbing-Skjern Municipality, Ringkøbing, Denmark; 3 Section of Freshwater Biology, Norwegian Institute for Water Research, Oslo, Norway; University of Yamanashi, Japan

## Abstract

We evaluated the restoration of physical habitats and its influence on macroinvertebrate community structure in 18 Danish lowland streams comprising six restored streams, six streams with little physical alteration and six channelized streams. We hypothesized that physical habitats and macroinvertebrate communities of restored streams would resemble those of natural streams, while those of the channelized streams would differ from both restored and near-natural streams. Physical habitats were surveyed for substrate composition, depth, width and current velocity. Macroinvertebrates were sampled along 100 m reaches in each stream, in edge habitats and in riffle/run habitats located in the center of the stream. Restoration significantly altered the physical conditions and affected the interactions between stream habitat heterogeneity and macroinvertebrate diversity. The substrate in the restored streams was dominated by pebble, whereas the substrate in the channelized and natural streams was dominated by sand. In the natural streams a relationship was identified between slope and pebble/gravel coverage, indicating a coupling of energy and substrate characteristics. Such a relationship did not occur in the channelized or in the restored streams where placement of large amounts of pebble/gravel distorted the natural relationship. The analyses revealed, a direct link between substrate heterogeneity and macroinvertebrate diversity in the natural streams. A similar relationship was not found in either the channelized or the restored streams, which we attribute to a de-coupling of the natural relationship between benthic community diversity and physical habitat diversity. Our study results suggest that restoration schemes should aim at restoring the natural physical structural complexity in the streams and at the same time enhance the possibility of re-generating the natural geomorphological processes sustaining the habitats in streams and rivers. Documentation of restoration efforts should be intensified with continuous monitoring of geomorphological and ecological changes including surveys of reference river systems.

## Introduction

The vast majority of European streams and rivers have been altered by human activities; thus, 95% of all riverine floodplains have been lost to agriculture and urbanization and river systems have been fragmented by thousands of major and minor dams influencing flow conditions and the longitudinal migration of organisms [1]. Many European rivers and streams are also characterized by high levels of organic pollution and nutrient enrichment from agriculture, and most river basins suffer from the combined impacts of pollution and elevated nutrient concentrations as well as physical habitat degradation [2], [3], [4]. The consequence of the past and contemporary degradation of European stream ecosystems is that the majority of streams fail to reach the “good ecological status” stipulated within the legislative context of the Water Framework Directive (WFD). Therefore, improvement of ecological status poses a key challenge to water managers, and there is an urgent need to implement cost effective mitigation measures and restoration projects to improve the ecological status of water bodies in Europe.

Since the 1970s policies adopted at local, regional, national and international levels have improved the water quality of streams, primarily through improved waste water treatment [5]. This improvement has however, only to a certain degree, been matched, by enhanced diversity of stream biota, the most likely explanation being poor physical conditions and improper management of rivers and their flows and insufficient time to re-colonize polluted reaches [6], [7], [8]. Since the 1990s focus has also been dedicated to improving in-stream habitat conditions through river rehabilitation/restoration across Europe and North America [9], [10], [11]. The dominant paradigm in river restoration has been rehabilitation of the physical system with primary focus on habitat structure and water flow to enhance habitat heterogeneity and biodiversity. Physical habitat restoration schemes typically work on a local scale and the measures implemented are usually introduction of gravel bars and patches of large woody debris (LWD) in small sections. At the intermediate scale restoration schemes aim to restore degraded river sections to their natural condition through re-meandering of entire sections of the river. The main objective of hydrological restoration is to obtain near natural hydrological conditions in entire catchments [12], [13]. However, by emphasizing only in-stream habitat heterogeneity the success of restoration efforts may be compromised if the geomorphological processes (e.g. interaction between flow regime and morphological units at different scales) behind the heterogeneity are not well understood [10], [14]. Thus, water managers may risk restoring the habitats to conditions that cannot be sustained on a longer temporal scale because fundamental physical laws governing the dynamic interaction between flow regime and geomorphology in a particular stream/river are inadequately considered. To sustain a heterogeneous environment capable of supporting diverse ecological communities, a key issue is to determine how natural streams and rivers are structured in terms of physical habitats and flow and sediment regime and how these three factors interact [10].

Knowledge of the dynamic linkages between forms and processes across different scales in natural streams and rivers is the key to understanding in-stream heterogeneity, and such understanding is essential to restore streams/rivers to natural conditions. Seen within the perspective of river ecology, or restoration ecology, the all-important issue is how spatial and temporal physical heterogeneity creates a range of niches and micro habitats for the biota in natural streams and how this heterogeneity can be recreated within a rehabilitation context [14], [15], [16]. The morphology of a river depends on catchment-scale controls (hydrology, geology), differences in channel patterns at reach scale (i.e. local slope, geology) and micro-scale variations in the structure and composition (flow and turbulence structure, bank material) of the river, factors that all vary over different time scales [17]. Hence, rivers experience a predictable morphological pattern at both reach and habitat scale (dominant bed type, entrenchment ratio, sinuosity, width to depth ratio and water surface slope), with topography and catchment geology playing at multiple scales a major role in structuring the habitats [18], [19].

Even though the number of river restorations has increased over the last several decades in both Europe and North America [11], [20], [21], studies providing conclusive empirical evidence of its effects are lacking [13]. A comprehensive review by Feld et al. [11] provided almost no evidence of a long term (+5 years) positive effect of river restoration on biotic communities. A very recent paper by Lorenz et al. [21] described, though, a longer term positive response of macrophytes to restoration measures. A similar conclusion to that of Feld et al. [11] was reached by Miller et al. [22] in a review of 24 case studies. Roni et al. [9] conclude that the lack of firm evidence is primarily a consequence of limited spatial and temporal resolution of data on physical habitats and biota. Long term monitoring and comparisons with reference stream systems, serving as restoration targets, are clearly needed by water policy managers and stakeholders in order to assess the socio-economic and ecological success of stream restoration schemes. The very limited evidence of links between restoration activities and improvement in ecological status constitutes a substantial problem for water managers when having to select appropriate measures as the costs involved can be very high [23].

The overall objective of the present study is to highlight important drivers of restoration success in lowland streams with Danish sites serving as examples. Denmark provides a unique opportunity for more conclusive restoration studies to be carried out, as several restoration projects have been implemented since the late 1980s in the Danish lowland landscape exhibiting limited spatial variability compared to the rest of the world [1], [24]. The Danish landscape consists primarily of soft sediments of glacial origin, ranging from sandy soils to loamy soils with up to 30% clay in a sandy matrix. Hence, on a global scale Denmark is geologically relatively homogeneous, allowing comparison of spatial variation in the physical environment among many sites. The Danish landscape is thus well suited for undertaking long term evaluation of restoration projects linking physical processes over a temporal scale ranging from years to decades with ecological recovery processes of invertebrate communities within a catchment context. We believe that our results may provide insight of general interest to both scientists and managers in both Europe and North America. To evaluate the effects of restoration on a longer time scale than previously, we evaluate restorations conducted in small Danish lowland streams involving both re-meandering and re-sectioning of the profile and in-stream habitat enhancement by gravel addition. We examine if physical habitats and macroinvertebrate communities in restored streams resemble those of channelized reaches and naturally meandering streams or whether a new ecological state has developed. We hypothesize that the physical conditions of restored streams will resemble those of natural streams and that this resemblance will be reflected in the macroinvertebrate communities.

## Methods

### Ethics statement

**Figure 1:**
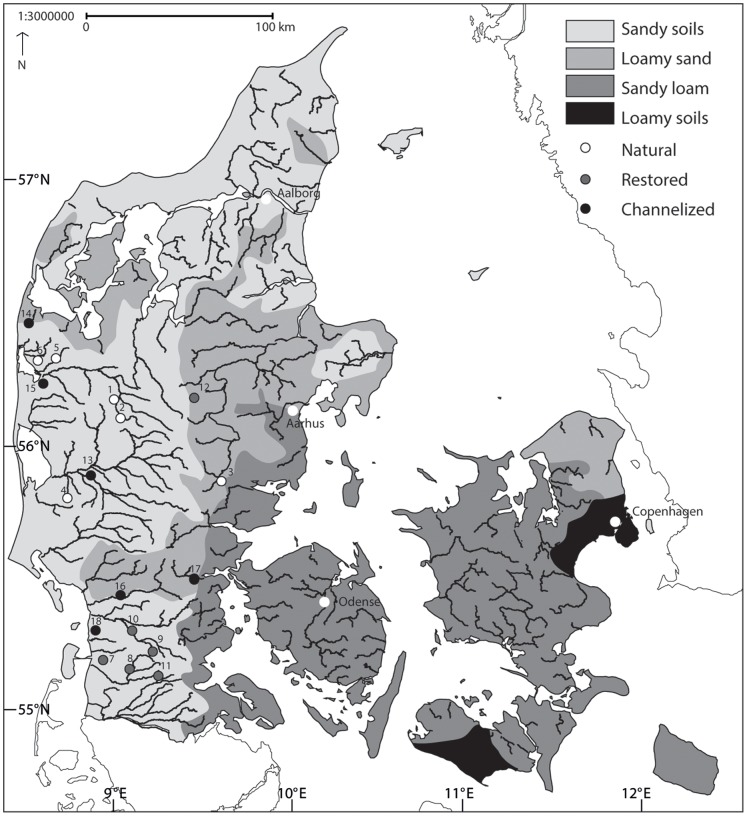
Location of the 18 stream reaches in Denmark. Natural streams (1–6); Restored streams (7–12); Channelized streams (13–18). UTM coordinates of the sites (UTM Zone 32, datum ED50). 1: Sunds Nørreå (N6231730; E496890), 2: Fjederholt (N6214415; E500939), 3: Linding (N6171439; E473283), 4: Gesager (N6190369; E543271), 5: Grydeå (N6243183; E471912), 6: Idom (N6243861; E468179), 7: Brøns (N6116409; E484998), 8: Lobæk (N6108125; E499423), 9: Surbæk (N6102701; E510372), 10: Jels (N6127631; E509606), 11: Gels (N6117435; E512790), 12: Lemming (N6233250; E532931), 13: Simmebæk (N6188424; E488854), 14: Fåre Mølleå (N6257940; E454624), 15: Madum (N6233919; E463860), 16: Hjortvad (N6137346; E494356), 17: Kongeå (N6141296; E519069), 18: Rejsby (N6121446; E483188).

All reaches were located on public watercourses, hence no permission were required to access the sites. All field sites are identified by UTM coordinates in Fig. 1. Protected and endangered species were carefully sorted from the samples in the field. Given the problems of identifying macroinvertebrates in the field, only the largest specimens were sorted alive before preservation. Smaller specimens could only be identified in the laboratory.

### Site selection

The study streams were all located in Jutland, Denmark (Fig. 1). The western part of Jutland remained ice-free during the last glaciation (ending 10,000 years ago) and the landscape is dominated by sandy soils developed on glacio-fluvial outwash plains and loamy sand soils on moraine hills from previous glaciations. The eastern part of Jutland was located close to the glacier margin and is mainly characterized by sub-glacial loamy moraine from the Weichsel glaciation. The dominant land use (app. 70%) in all catchments is agriculture with smaller areas of forest, heath land and wetlands (Table S1). Mean annual precipitation varies between 900 and 1000 mm and the hydrological regime is dominated by groundwater during summer, whereas increased precipitation and lower evaporation result in higher discharge during winter. The hydrological regime is also affected by drainage of agricultural areas in the catchments, and even naturally meandering streams are thereby also impacted by land use changes, leading to changes in hydrological regime and sediment dynamics.

Eighteen reaches were selected, each from a different stream to avoid interdependence (stream width 2–5 m, depth app. 0.50–0.70 m; Table 1) and irrespective of catchment geology. Streams were only included in the surveys, which were conducted in April and May 2002, if no point sources of nutrients and pollutants (fish farms, waste water treatment plants, lakes, reservoirs etc.) occurred upstream the surveyed reach. The reaches were 100 m long and covered approximately 5 riffle-pool sequences depending on stream width. Six streams were in a near natural meandering state with little physical alterations; hereafter referred to as “Natural” streams. Six were channelized and 6 were former channelized reaches that had been re-meandered minimum 3 years prior to the investigation (Fig. 1). The re-meandered stream reaches were selected to include a buffer of at least 200 m restored reach upstream of the study reach.

**Table 1:** Physical characteristics of the 18 streams.

	Stream type		
	Natural	Channelized	Restored
Catchment area (km^2^)	61±32	44±22	81±28
Slope (‰)	1.6±0.6(ab)	0.6±0.3(b)	1.9±1.1(a)
Substrate heterogeneity	0.32±0.07	0.36±0.20	0.41±0.10
Current velocity (m/s)	0.34±0.04(a)	0.26±0.05(b)	0.30±0.04(ab)
Current velocity_CV_ (%)	27±4(a)	14±5.38(b)	18±5(b)
Width (cm)	392±56	360±137	493±121
Width_CV_ (%)	18±7(a)	7±1(b)	12±5(ab)
Depth (cm)	52±18	46±15	47±15
Depth_CV_ (%)	44±3(ab)	34±7(b)	50±9(a)

Mean values are presented along with standard deviations (SD). Letters indicate significant differences among stream types using one-way ANOVA and pair-wise Bonferroni corrected *post hoc* tests.

Field work was conducted over a 40 day period in April and May 2002. In order to minimize the effects of high flow event field work were only carried if no precipitation had occurred in the previous 7 days prior to the sampling. One week was omitted from sampling in order to reduce the risk of influence from heavy rain showers.

### Catchment and river corridor data

Data on catchment geology and land use were extracted from the national GIS data base using ArcGIS (ArcGIS Desktop 10, ESRI). River corridor land use was also extracted from a buffer covering a width of 50 meters on each side of the stream and extending 1 kilometer up- and downstream from the field site. This analysis was also conducted in the ArcGIS environment.

In the re-meandered streams, pebble and gravel and to a lesser degree stone substrate had been added to the stream bed to increase habitat diversity. The banks had been re-profiled and, in some cases, the bed level had been raised to increase hydrological interactions with the floodplain. All restoration measures were aimed at creating a more heterogeneous and hence natural stream reach.

### Water chemistry

In order to characterize the water chemistry of the sites and to ensure that the water chemistry did not affect biological communities, nine chemical variables were analyzed in the laboratory. Biological oxygen demand over five day (BOD_5_) was measured according to Danish Standard 1899∶1 [25]. pH was measured on a PHM240 pH-meter and alkalinity was determined by Gran titration on 100 mL subsamples of stream water [26]. Ferro-iron (Fe^2+^), total-N, NH_4_^2+^, NO_3_^−^, total-P and PO_4_^2−^ (all mg/L) were measured according to Danish Standards, DS 219 [27], DS 221 [28], DS 223 [29], DS 292 [30] and DS 291 [31], respectively.

### Physical habitats

The physical habitats were measured in 120 plots (25×25 cm) placed side-by-side covering the entire width of the stream in 10 to 12 equally spaced cross sectional transects along the 100 m reach. Water depth was measured to nearest cm in the middle of each plot and mean depth was subsequently calculated. Stream width was measured from bank to bank at each transect and the mean width of the stream reach was calculated. In order to quantify the variation in stream reach dimensions, the coefficient of variation of depth and width was calculated [32].

The dominant substrate type in each plot was categorized according to a modified Wentworth scale [33] as: cobble (>64 mm diameter), pebble/gravel (2–64 mm), sand (0.1–2 mm), silt/clay (<0.1 mm, inorganic particles, usually with a compact structure) and mud (<0.1 mm, a mixture of inorganic particles and organic debris (FPOM), typically brown or black, loosely structured). The relative frequency of the various substrate types was calculated from these recordings. Substrate heterogeneity (SH) was quantified from the spatial distribution of substrate types according to Pedersen et al. [24].

The average current velocity and the velocity heterogeneity were characterized by measuring the current velocity in four different depths in five vertical profiles equally spaced across the stream at the downstream end of the reach). Velocities were measured with a propeller current meter (Kleinflügel, OTT Instruments).

### Biological sampling of macroinvertebrates

Macroinvertebrates were sampled using a stratified random sampling methodology. Two main meso-habitats were identified: the edge habitat, located close to the bank having current velocities below 0.1 m/s, and a riffle/run habitat typically located in the center of the stream with current velocities exceeding 0.1 m/s.

A total of six macroinvertebrate samples were collected at each reach. Within each of the two meso-habitats 3 surber (500 cm^2^; 500 µm mesh size) samples were collected by disturbing the upper 5 cm of the stream bed. The sampling locations were selected randomly among the 120 surveyed habitat plots at each reach. All samples were preserved in 70% ethanol and transported to the laboratory for sorting and identification. Macroinvertebrates were identified to species level except for dipterans, which were identified to sub-family level, and oligochaetes, which were identified to sub-class level. Protected and endangered species were carefully sorted from the samples in the field. Given the problems of identifying macroinvertebrates in the field, only the largest specimens were sorted alive before preservation. Smaller specimens could only be identified in the laboratory.

### Statistical analyses

For each sample macroinvertebrate community structure and diversity were expressed using several metrics. Species richness and total invertebrate abundance and total abundance of Ephemeroptera, Plecoptera, Trichoptera and Coleoptera (EPTC), Shannon-Wiener diversity (H’), were calculated for each sample [34]. All metrics were log-transformed prior to any further analyses to satisfy assumptions of normality. To test for differences among stream types (natural, restored, channelized) a nested analysis of variance (ANOVA) was used, where samples and streams were nested in type. This allowed us to test for differences in macroinvertebrate metrics among types and at the same time correcting for repeated sampling within types [35].

Macroinvertebrate community composition at the 18 sites was analyzed by means of Detrended Correspondence Analyses (DCA) using PC-ORD version 6 (MjM Software) and then related to environmental variables by means of Spearman rank correlation analysis. All the above mentioned macroinvertebrate metrics were also calculated for each reach by pooling the 6 samples. Using type (natural, restored or channelized) as a co-variate, a Spearman rank correlation analysis between reach-scale physical parameters and macroinvertebrate community variables was performed in order to elucidate the effects of channelization and restoration on community structure [35]. Significant relationships between physical parameters and macroinvertebrate metrics were further analyzed and quantified using ANOVA analysis and subsequently linear regression analysis. Residuals of all developed relationships were tested for normality to satisfy the assumptions of regression analysis [36]. Additional ANOVA analyses were carried in order to test for possible confounding factors. Factors included: water chemistry, catchment geology, river system location and years since restoration.

## Results

### Physical habitats

**Figure 2:**
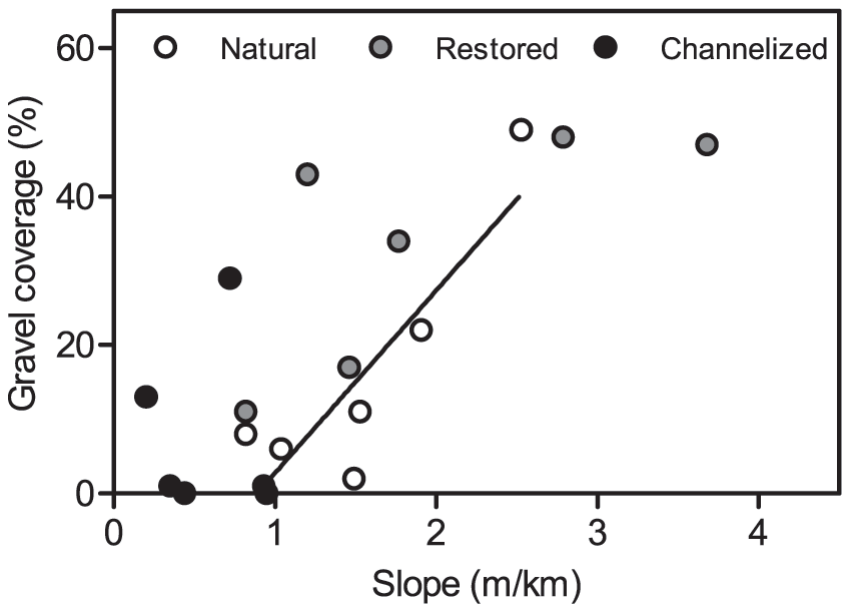
Relationship between stream bed slope and gravel coverage in the 3 stream types. The solid regression line describes the relationship in natural streams.

**Figure 3:**
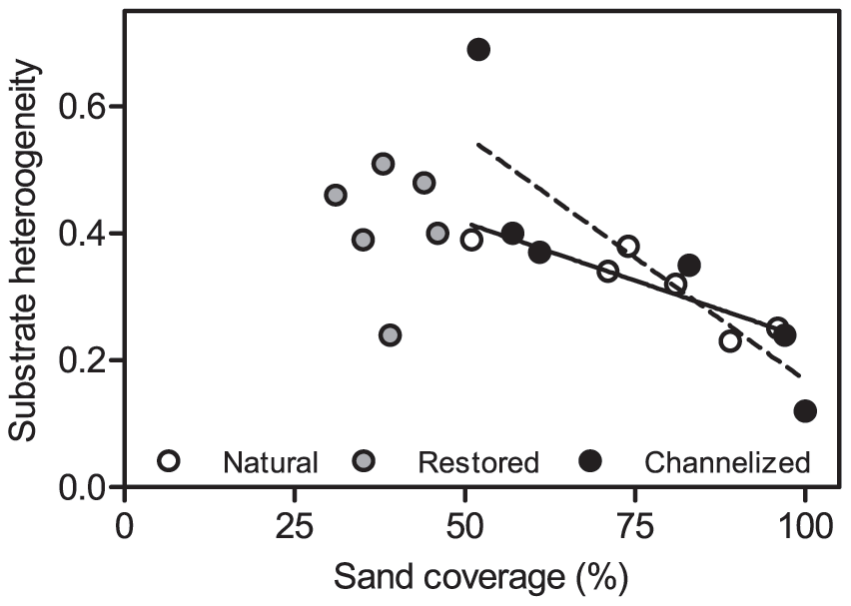
Relationship between stream bed sand coverage and substrate heterogeneity in the 3 stream types. The solid regression line describes the relationship in natural streams and the dotted line is the fitted line for channelized streams.

The composition of the stream bed substrate varied significantly among stream types, cobble and pebble being significantly dominant in restored streams and sand in natural and channelized streams (Table S2). A significant empirical relationship existed between stream bed slope and coverage of pebble and gravel in natural streams (R^2^ = 0.76; p = 0.025; Fig. 2). In contrast the relationship in both channelized and restored streams were not significant; R^2^ = 0.01 (p = 0.87) and R^2^ = 0.52 (p = 0.11), respectively. In natural streams the heterogeneity of the stream bed substrate decreased with a linear increase in the coverage of sand as expected (R^2^ = 0.76; p = 0.025; Fig. 3). Also in the channelized streams substrate heterogeneity was inversely related to the coverage of sand (R^2^ = 0.73; p = 0.031; Fig. 3), while the regression line parameters differed from those of the natural streams. This relationship between substrate heterogeneity and sand cover was not detected in the restored streams (Fig. 3), indicating a de-coupling of natural physical processes structuring the stream bed composition; the de-coupling arise from the addition of gravel and stones to the restored stream beds.

Marked differences were also found in the heterogeneity of stream dimensions and current velocities. In natural streams width variation was significantly higher (18%) than in channelized streams (7%). In the restored streams some of the natural variation in width had been recreated through re-meandering, variation being intermediate (Table 1). Depth varied most markedly in the restored streams and was significantly higher than in the channelized streams. Natural streams exhibited the significantly highest variation in the flow environment, but velocity variation in restored reaches was more similar to that of channelized reaches, indicating a failure of restoration to restore a natural flow environment (Table 1).

### Macroinvertebrate communities

**Figure 4:**
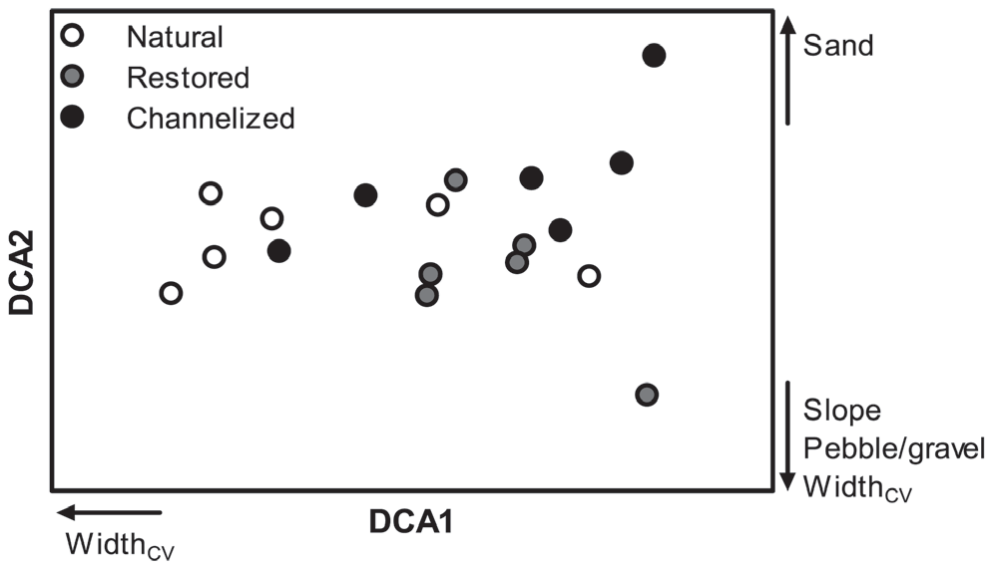
DCA ordination of macroinvertebrate communities in natural, channelized and restored stream reaches. Environmental parameters significantly (Spearman rank correlation, p<0.05) correlated with in-stream physical characteristics are also shown.

The DCA analysis of the macroinvertebrate communities revealed a distinction between stream types. The natural streams were located along the entire DCA axis 1 gradient and showed pronounced variation in width and large within-group variations in species composition (Fig. 4). Natural streams showed little variation in DCA axis 2 values. Coverage of pebble/gravel and slope were correlated with DCA axis 2, which is reflected in the species distributions of both channelized and restored streams. This reflects differences in species distribution among the stream types. A total of 129 taxa were encountered across the 18 sites (Table S3). When contemplating the 10 most dominant taxa in the three stream types little variation appeared (Table S4). The bulk of the community does not differ among stream groups; however, there are indications of differences in species composition, which probably reflects differences in the physical environment. Taxa groups normally associated with coarse grained substrates (Ephemeroptera, Plecoptera, Trichoptera and Coleoptera) are located and low and intermediate DCA axis 2 scores in the species plot (Fig. S1).

**Figure 5:**
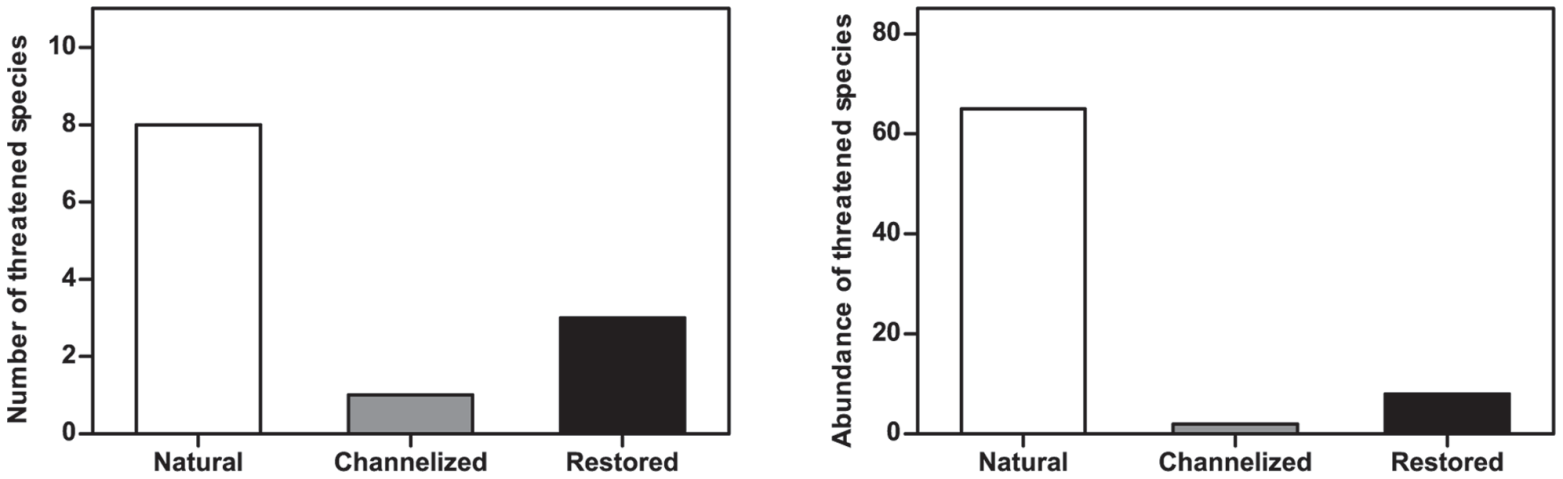
Number of threatened species and abundance according to stream type. In total, 75 individuals of 9 threatened species were found in the 18 streams.

Macroinvertebrate community metrics varied negligibly among stream types, i.e. taxonomic richness, abundance, evenness and diversity measures showed no significant differences (Table 2). When combining the DCA analysis and results from Table 2, it is evident that the community metrics used reveal no effects of channelization and restoration; however, endangered species occurred more frequently in the natural streams. Moreover, endangered species occurred in 5 out of 6 natural streams but only in 1 restored and 2 channelized streams (Fig. 5), reflecting the sensitivity of these species to channelization and thus the limited possibility of their re-colonization after restoration due to habitat modifications and hence low availability of adequate habitats along the stream.

**Table 2:** Macroinvertebrate community metrics in the three stream types.

	Stream type		
	Natural	Channelized	Restored
Taxa richness	47.2±7.2	36.5±12.1	43.8±6.5
Abundance	2828±1083	1873±1460	2683±707
Shannon diversity (H’)	2.28±0.26	2.25±0.27	2.19±0.24
Evenness	0.59±0.06	0.64±0.08	0.58±0.06
EPTC taxa	24.5±2.2	24.3±2.8	24.5±4.7
ETPC abundance	845±268	423±145	787±540

Mean values are presented along with standard deviations (SD).

**Figure 6:**
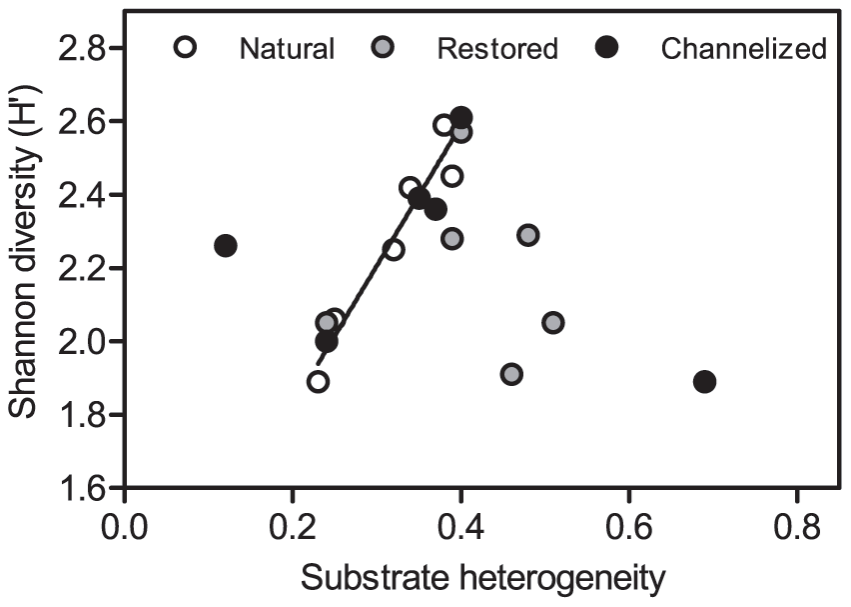
Relationship between substrate heterogeneity and macroinvertebrate community diversity for the 3 stream types. The solid regression line shows the significant relationship in natural streams.

It is generally assumed that high habitat heterogeneity is matched by high species diversity. Our study provided no conclusive evidence for the existence of such a relationship across all study sites combined. Instead, a relationship was established between habitat heterogeneity measured as substrate heterogeneity and species richness only in natural streams (R^2^ = 0.93; p = 0.0021; Fig. 6). The same relationship did not emerge along either the channelized or the restored reaches despite the fact that variation in substrate heterogeneity was lower in natural streams compared to channelized and restored streams.

### Possible confounding factors

We examined several possible confounding influences on our analyses: time since restoration, water chemistry, catchment geology, river system location, river corridor land use and variation in stream size. The analysis revealed no significant influence of river system location despite a tendency towards location of restored stream in the southern part of Jutland (ANOVA, p>0.05). The study streams were restored over a 10-year period and differences in recovery time may potentially have influenced the results. Using time since restoration as a co-variate, all the developed relationships were analyzed for this confounding factor, but no effect was demonstrated (ANOVA, p>0.05). No significant effects of catchment geology and land use on the results was found (ANOVA, p>0.05; Table S1). River corridor land use varied among the groups. The natural sites were dominated by wetlands and natural riparian areas, whereas channelized and restored streams were dominated by agricultural land use (Table S5). The water chemistry variables showed no significant variation among the groups (one way ANOVA, p>0.05; Table S6) and water chemical stress was therefore assumed to be uniformly distributed among the stream types. The natural, channelized and restored streams used in the present study were of similar size. No significant differences occurred in the catchment area or regarding stream width and depth among the stream types (Table 1). The stream bed slope varied significantly between the stream types; the highest slopes appearing in the restored streams (mean = 1.9 m/km) and the lowest in the channelized streams (mean = 0.6 m/km). The natural streams were characterized by intermediate slopes.

Correlation coefficients among the physico-chemical parameters are given in the supplementary material (Table S7). There are few correlations among the parameters when analyzed across the entire dataset, probably reflecting the effects of channelization and restoration. Coarse substrate coverage increased with increasing catchment area and was inversely correlated with coverage of sand, as indicated by the results in Fig. 3. The variation in velocity increased with increasing variation in width, probably due to enhanced physical variation in heterogeneous streams.

## Discussion

### Restoration effect studies

The effects of restoring, re-meandering or re-habilitating river and streams have been documented in numerous studies conducted primarily in Western Europe and North America over the past 30–40 years [11], [20]. The results of most studies are, however, inconclusive. In a recent review of 345 projects Roni et al. [9] concluded that: “… firm conclusions …were difficult to make because of the limited information provided on physical habitat, water quality, and biota …”. A similar conclusion was reached by Palmer et al. [37]. The lack of clear results is partly attributed to inadequate pre- and post-project monitoring, which is often neglected by water managers [13], [22], [38], [39], and partly to the focus of most re-habilitation schemes on reach scale and lacking consideration of catchment processes [40]. With this in mind we evaluated the success of restoration in 6 streams by comparing physical habitats and the response of macroinvertebrates to restoration in channelized and natural streams. We found significant effects of restoration on some physical habitat parameters and on the interactions between stream habitat heterogeneity and macroinvertebrate diversity.

### Physical habitats

We found significant changes of the physical habitats in restored streams compared to natural (reference) and channelized streams. Pebble and gravel dominated the substrate composition in the restored streams, whereas sand was the most prominent substrate in the channelized and natural streams. This clearly shows that too much emphasis is given to gravel bed restoration in this setting, and the pebble/gravel coverage is significantly higher than in natural streams. Little effort is devoted to balancing substrate composition and stream slope. The stream bed slope varied significantly among the three stream types; the highest slopes occurring in the restored streams and the lowest in the channelized streams. This may seem contradictory but is a consequence of differences in catchment slope rather than of past effects of river regulation. The key to natural morphology and biodiversity in restored rivers is thus to ensure a balance between stream slope (or rather stream power) and substrate [41]. Channelization and dredging remove coarse substrate from the stream bed. The physical and biological impacts of this are well documented [42], [43]. Despite this it appears as if water managers install too much coarse substrate, affecting colonization and reestablishment of the macroinvertebrate community and, in consequence, community composition and long term ecosystem processes in the restored streams [11]. The time scale and set-up of our study did not allow us to quantify ecosystem processes (e.g. decomposition of organic matter) which have the potential of becoming a useful indicator of ecosystem change [11], [44], [45], [46].

The restoration work carried out in Denmark as well as in the rest of Europe and North America primarily rests on the principle of natural channel design (NCD) [18], [47]. This principle, and hence the restoration efforts, is primarily used at the reach scale [48]. NCD is basically a static design principle and critics argue that the form-based system ignores the processes in alluvial streams where form and substrate continuously adjust to varying water and material inputs [49], [50] – in other words, the principle oversimplifies the physical/geomorphological processes in the streams [49], [51]. In-stream heterogeneity measured as the coefficient of variation in width, depth and velocity was only partly re-established by the stream restoration measures. At reach scale no significant differences appeared in substrate heterogeneity. Our results indicate that the natural functioning of the geomorphological processes is affected both by channelization and by restoration and, moreover, that restoration does not re-establish the natural functioning of the processes at the time-scale (3+ years) studied here. Studies over longer periods of time are required to document the recovery of natural physical processes in restoration schemes where in-stream substrate composition has been altered beyond natural conditions. In natural streams the recorded relationship between slope and pebble/gravel coverage indicated coupling between energy and substrate characteristics; the more energy the more coarse substrate. This relationship was probably not present in the channelized streams due to dredging and in restored streams due to the placement of large amounts of pebble/gravel, which distorts the natural relationship. This may on a longer time scale affect other geomorphological (sediment transport, shear stress interactions with stream bed) and biological processes (e.g. plant recolonization) in the streams and hence possibly also the recovery of biotic communities to a natural state. Moreover, the absence of a relationship between sand coverage and substrate heterogeneity in restored streams is a clear indicator of the disruption of natural dynamic processes in restored streams.

### Macroinvertebrates

One indisputable result emerged from our study – namely the direct link between substrate heterogeneity and macroinvertebrate diversity in natural streams, a similar relationship being non-existing in channelized and restored streams. This result supports the general ecological hypothesis as well as the specific stream ecology hypothesis that biodiversity is closely linked with habitat heterogeneity [52], [53], [54], [55], [56], [57]. Habitat heterogeneity is, however, loosely defined [10], rendering comparisons of results difficult. In our study substrate heterogeneity is used as a surrogate for habitat heterogeneity. Mixed results have been reported for correlating in-stream heterogeneity and diversity in stream ecosystems [10]. Pedersen et al. [15] found an increase in invertebrate community diversity and evenness 3 years after restoring a large lowland river. Similarly, O’Connor [58] recorded an increase in habitat diversity and species richness from large woody debris in a study in Australia. The meta-analysis by Miller et al. [22] indicated that in some cases increased habitat complexity is matched by increases in macroinvertebrate community metric scores. Jähnig et al. [59] also reported the existence of a relationship between macroinvertebrate community and habitat diversity.

Despite significant differences in physical habitat conditions, macroinvertebrate taxonomic richness, abundance and diversity showed a similar lack of response channelized and restored reaches. A similar absence of response was reported from at meta-analysis study of 24 projects by Miller et al. [22]. Ernst et al. [40] found that only one macroinvertebrate metric responded to restoration in small forested headwater streams in the Catskill Mountains in New York State. Such a lack of response is consistent with the results of numerous other studies recording little or no response of macroinvertebrates to restoration. Lepori et al. [60] concluded that local scale restoration had little effect on macroinvertebrate communities compared to watershed scale factors. In a meta-analysis of stream restoration projects from 1975 to 2008, Palmer et al. [10] found that only 2 of 78 restoration projects generated increases in macroinvertebrate diversity.

Corroborating the conclusions reached by Lepori et al. [60], other studies have revealed that the positive effects of restoration can be short-lived because of catchment-scale impacts. Thus, Moerke and Lamberti [61] found that restoration of a channelized stream in the Midwest led to immediate improvement of habitat quality, but the improvement became less noticeable three years later because of continued high rates of erosion in the watershed. Similarly, Ernst et al. [40] concluded that catchment-scale factors were more important than restoration efforts in structuring the macroinvertebrate community. The reach by reach approach to restoration taken in our study did not address upstream stressors or catchment scale issues that may continuously affect in-stream biodiversity. We did, though, select our stream reaches in a way to ensure that chemical stress was at comparable levels for all reaches irrespective of stream type (natural, channelized or restored).

Effect studies of river restoration in agricultural landscapes are always subject to influence from confounding factors, such as the higher intensity of agriculture in the riparian zones of the channelized and restored streams compared with natural streams. This is an inherent problem in this type of studies as streams in lowland areas have been channelized to improve draining of riparian areas to create suitable conditions for farming. However, our substrate data from the restored streams suggest no major impacts of siltation by fine sediments or changed hydrology (erosion) that can be related back to riparian land use. Regarding the biota it is not possible to separate any additive effects of riparian land-use on community structure. Although studies have been able to link arable land-use with negative ecological status of rivers [4], in our case “agriculture” will include less intensive forms of farming (e.g. pastures) making it unlikely that riparian land-use should be the major driver of the patterns we observe.

## Conclusions

Two main conclusions can be drawn from our work. Firstly, river restoration, as practiced in Denmark today, does not restore streams to natural conditions *per se*. Habitat diversity is somewhat enhanced compared to channelized reaches in terms of width and depth variations, but the addition of large quantities of gravel and pebble to the restored streams skews the substrate composition in a non-natural direction. Large quantities of coarse substrate will likely influence macroinvertebrate colonization and hence community composition. Secondly, relationships between slope, substrate composition, substrate heterogeneity and macroinvertebrate diversity are affected by the excessive use of gravel/pebble in these restored streams, potentially influencing geomorphologic and biological processes.

## Recommendations

Our results clearly suggest that restoration schemes should aim at restoring natural structures and enhancing the possibility of re-generating the natural geomorphological processes sustaining the habitats in streams and rivers. The excessive use of pebble and gravel should be abandoned and replaced by generating a more natural substrate distribution, mimicking those of reference streams. More investigations should be carried out with focus on developing biological indicators of habitat improvements [62], [63]. Macroinvertebrates are an important organism/functional group in streams, but their mixed response to restoration and habitat improvement suggests than other organism groups should be included. Moreover, more emphasis should perhaps be given to developing functional and process based metrics. Even though documentation of restoration efforts is plentiful, the quality of the data is somewhat questionable as suggested by Palmer et al. [10] and Miller et al. [22]. Scientists must keep monitoring the effects and work together with water managers in an effort to increase monitoring activities both before and after restoration. Water managers and scientists need to collaborate on putting restoration schemes into the right perspective. Hence, catchment-scale restoration plans and schemes acting beyond the reach are important to obtain scientific documentation of river restoration on a larger scale.

## Supporting Information

Figure S1 **DCA Species plot from all 18 stream reaches.** Abbreviations: Hydr.ind : Hydracarina indet.; Oreo.san : *Oreodytes sanmarkii*; Elmi.aen : *Elmis aenea*; Limn.vol : *Limnius volckmari*; Ouli.sp : *Oulimnius* sp.; Orec.vil : *Orectochilus villosus*; Hali.sp : *Haliplus* sp.; Elod.m.g : *Elodes minuta* gr.; Athe.ibi : *Atherix ibis*; Cera.ind : Ceratopogoninae indet; Chir.ind : Chironominae indet; Orth.ind : Orthocladinae indet; Prod.ind : Prodiamesinae indet; Tany.ind : Tanypodinae indet; Empi.ind : Empididae indet; Hexa.ind : Hexatominae indet; Pedi.ind : Pediciinae indet; Ptyc.sp : *Ptychoptera* sp.; Simu.ind : Simuliidae indet; Ostr.ind : Ostracoda indet.; Baet.nig : *Baetis niger*; Baet.rho : *Baetis rhodani*; Baet.sp : *Baetis* sp.; Baet.ver : *Baetis vernus*; Cent.lut : *Centroptilum luteolum*; Caen.riv : *Caenis rivulorum;* Ephe.ign : *Ephemerella* ignita; Ephe.sp : *Ephemerella* sp.; Ephe.dan : *Ephemera* danica; Hept.fus : *Heptagenia fuscogrisea*; Hept.sul : *Heptagenia sulphurea*; Lept.mar : *Leptophlebia marginata*; Para.sp : *Paraleptophlebia* sp.; Para.sub : *Paraleptophlebia submarginata;* Acro.lac : *Acroloxus lacustris;* Ancy.flu : *Ancylus fluviatilis;* Lymn.per : *Lymnaea peregra*; Phys.fon : *Physa fontinalis*; Velia.sp : *Velia* sp.; Erpo.oct : *Erpobdella octoculata;* Glos.com : *Glossiphonia complanata;* Helo.sta : *Helobdella stagnalis;* Hydra.sp : *Hydra* sp.; Pisi.sp : *Pisidium* sp.; Asel.aqu : *Asellus aquaticus;* Gamm.pul : *Gammarus pulex;* Sial.ful : *Sialis fuliginosa;* Sial.lut : *Sialis lutaria;* Olig.ind : Oligochaeta indet.; L.fu.di : *Leuctra fusca/digitata;* Amph.sp : *Amphinemura* sp.; Nemo.cin : *Nemoura cinerea;* Nemo.sp : *Nemoura* sp.; Isop.dif : *Isoperla difformis;* Brac.mac : *Brachycentrus maculatus*; Hydr.pel : *Hydropsyche pellucidula*; Hydr.sil : *Hydropsyche siltalai;* Lepi.hir : *Lepidostoma hirtum;* Athr.sp : *Athripsodes* sp.; Anab.ner : *Anabolia nervosa;* Eccl.dal : *Ecclisopteryx dalecarlica;* Hale.rad : *Halesus radiates;* Hale.sp : *Halesus* sp.; Limn.lun : *Limnephilus lunatus;* Limn.rho : *Limnephilus rhombicus;* Pota.cin : *Potamophylax cingulatus;* Pota.lat : *Potamophylax latipennis;* Pota.sp : *Potamophylax* sp.; Plec.con : *Plectrocnemia conspersa*; Poly.fla : *Polycentropus flavomaculatus;* Rhya.nub : *Rhyacophila nubile*; Rhya.sp : *Rhyacophila* sp.; Noti.cil : *Notidobia ciliaris*; Seri.per : *Sericostoma personatum*; Duge.gon : *Dugesia gonocephala*.
(TIF)

Table S1 **Catchment geology and land use characteristics of the natural, channelized and restored streams.** Mean values are presented along with standard deviations (SD). P-values for the one-way ANOVA analyses on arc sine transformed data are also shown.
(DOCX)

Table S2 **In-stream substrate composition.** Mean values are presented along with standard deviations (SD). Upper case letters indicate significant differences among stream types using one-way ANOVA and pair-wise Bonferroni corrected *post hoc* tests.
(DOCX)

Table S3 **Benthic macroinvertebrate taxa encountered across the 18 sites included in the survey.**
(DOCX)

Table S4 **Benthic macroinvertebrates –10 common taxa in the different stream types.** Mean abundance (per m^2^) is presented along with taxonomic names.
(DOCX)

Table S5 **River corridor land use characteristics of natural, channelized and restored streams.** Mean values are presented along with standard deviations (SD).
(DOCX)

Table S6 **Water chemistry characteristics of the natural, channelized and restored streams.** Mean values are presented along with standard deviations (SD).
(DOCX)

Table S7 **Spearman rank correlation coefficients among the physico-chemical parameters from the stream reaches.** P values are also presented in brackets (N = 18). Significance levels: *: 0.05; **: 0.01; ***:0.001
(DOCX)
