# Correction: Re-Meandering of Lowland Streams: Will Disobeying the Laws of Geomorphology Have Ecological Consequences?

**DOI:** 10.1371/journal.pone.0118939

**Published:** 2015-03-05

**Authors:** The PLOS ONE Staff

An incorrect version of Fig. 1 was published. Please see the correct Fig. 1 here.

**Fig 1:**
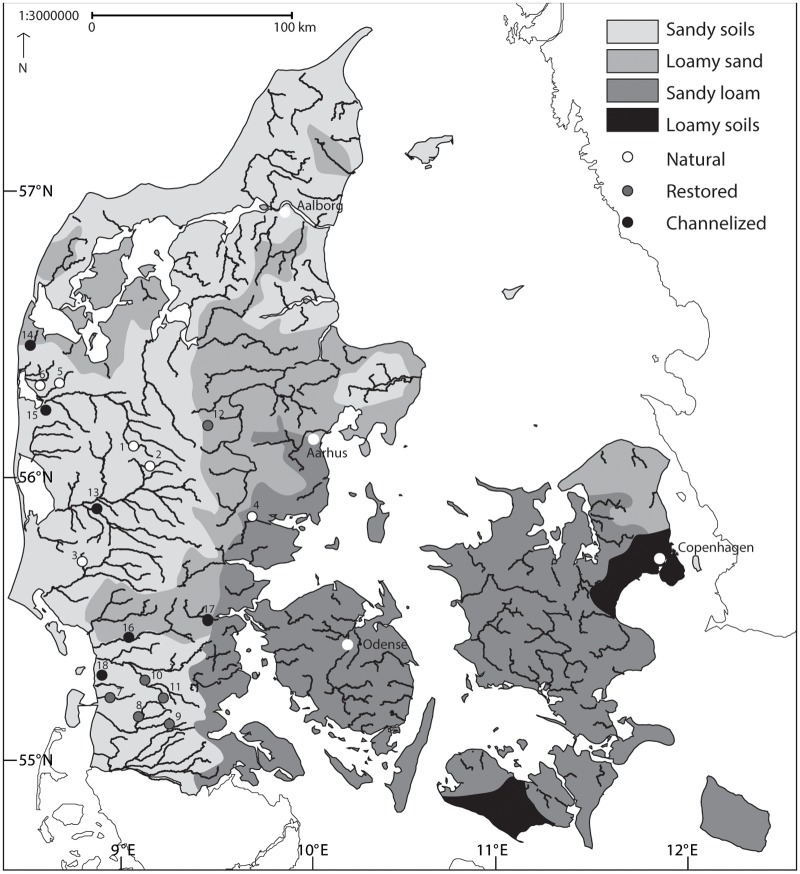
Location of the 18 stream reaches in Denmark. Natural streams (1–6); Restored streams (7–12); Channelized streams (13–18). UTM coordinates of the sites (UTM Zone 32, datum ED50). 1: Sunds Nørreå (N6231730; E496890), 2: Fjederholt (N6214415; E500939), 3: Linding (N6171439; E473283), 4: Gesager (N6190369; E543271), 5: Grydeå (N6243183; E471912), 6: Idom (N6243861; E468179), 7: Brøns (N6116409; E484998), 8: Lobæk (N6108125; E499423), 9: Surbæk (N6102701; E510372), 10: Jels (N6127631; E509606), 11: Gels (N6117435; E512790), 12: Lemming (N6233250; E532931), 13: Simmebæk (N6188424; E488854), 14: Fåre Mølleå (N6257940; E454624), 15: Madum (N6233919; E463860), 16: Hjortvad (N6137346; E494356), 17: Kongeå (N6141296; E519069), 18: Rejsby (N6121446; E483188.
