# Supplementary figures and images for: Re-Meandering of Lowland Streams: Will Disobeying the Laws of Geomorphology Have Ecological Consequences?
Source: PLoS One. 2014 Sep 29;9(9):e108558. doi: 10.1371/journal.pone.0108558 (PMC4180926; doi:10.1371/journal.pone.0108558)

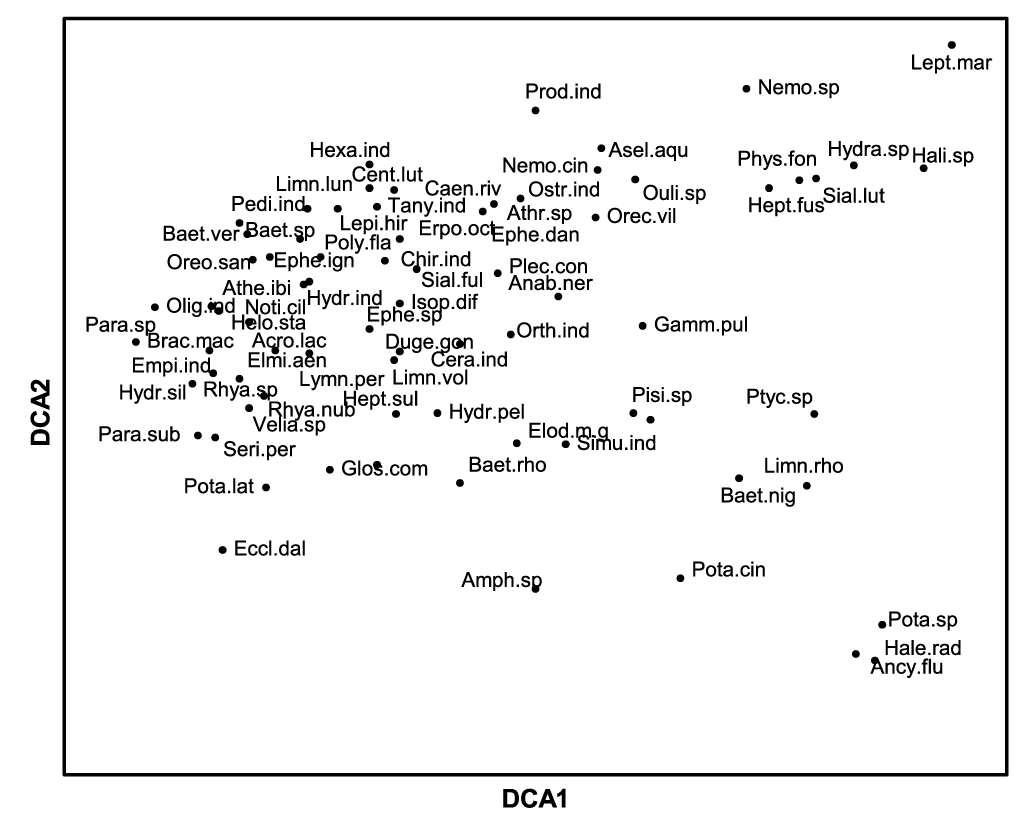

Supplement: Figure S1 — DCA Species plot from all 18 stream reaches. Abbreviations: Hydr.ind : Hydracarina indet.; Oreo.san : Oreodytes sanmarkii; Elmi.aen : Elmis aenea; Limn.vol : Limnius volckmari; Ouli.sp : Oulimnius sp.; Orec.vil : Orectochilus villosus; Hali.sp : Haliplus sp.; Elod.m.g : Elodes minuta gr.; Athe.ibi : Atherix ibis; Cera.ind : Ceratopogoninae indet; Chir.ind : Chironominae indet; Orth.ind : Orthocladinae indet; Prod.ind : Prodiamesinae indet; Tany.ind : Tanypodinae indet; Empi.ind : Empididae indet; Hexa.ind : Hexatominae indet; Pedi.ind : Pediciinae indet; Ptyc.sp : Ptychoptera sp.; Simu.ind : Simuliidae indet; Ostr.ind : Ostracoda indet.; Baet.nig : Baetis niger; Baet.rho : Baetis rhodani; Baet.sp : Baetis sp.; Baet.ver : Baetis vernus; Cent.lut : Centroptilum luteolum; Caen.riv : Caenis rivulorum; Ephe.ign : Ephemerella ignita; Ephe.sp : Ephemerella sp.; Ephe.dan : Ephemera danica; Hept.fus : Heptagenia fuscogrisea; Hept.sul : Heptagenia sulphurea; Lept.mar : Leptophlebia marginata; Para.sp : Paraleptophlebia sp.; Para.sub : Paraleptophlebia submarginata; Acro.lac : Acroloxus lacustris; Ancy.flu : Ancylus fluviatilis; Lymn.per : Lymnaea peregra; Phys.fon : Physa fontinalis; Velia.sp : Velia sp.; Erpo.oct : Erpobdella octoculata; Glos.com : Glossiphonia complanata; Helo.sta : Helobdella stagnalis; Hydra.sp : Hydra sp.; Pisi.sp : Pisidium sp.; Asel.aqu : Asellus aquaticus; Gamm.pul : Gammarus pulex; Sial.ful : Sialis fuliginosa; Sial.lut : Sialis lutaria; Olig.ind : Oligochaeta indet.; L.fu.di : Leuctra fusca/digitata; Amph.sp : Amphinemura sp.; Nemo.cin : Nemoura cinerea; Nemo.sp : Nemoura sp.; Isop.dif : Isoperla difformis; Brac.mac : Brachycentrus maculatus; Hydr.pel : Hydropsyche pellucidula; Hydr.sil : Hydropsyche siltalai; Lepi.hir : Lepidostoma hirtum; Athr.sp : Athripsodes sp.; Anab.ner : Anabolia nervosa; Eccl.dal : Ecclisopteryx dalecarlica; Hale.rad : Halesus radiates; Hale.sp : Halesus sp.; Limn.lun : Limnephilus lunatus; Limn.rho : Limnephilus rhombicus; [file pone.0108558.s001.tif]
