# Supplementary material for: Re-Meandering of Lowland Streams: Will Disobeying the Laws of Geomorphology Have Ecological Consequences?
Source: PLoS One. 2014 Sep 29;9(9):e108558. doi: 10.1371/journal.pone.0108558 (PMC4180926; doi:10.1371/journal.pone.0108558)
Supplement: Table S1 — Catchment geology and land use characteristics of the natural, channelized and restored streams. Mean values are presented along with standard deviations (SD). P-values for the one-way ANOVA analyses on arc sine transformed data are also shown. (DOCX) [file pone.0108558.s002.docx]

|  | Stream type | | |  |
| --- | --- | --- | --- | --- |
|  | Natural  (n=6) | Channelized  (n=6) | Restored  (n=6) | p-value |
| Loamy till | 14 ± 19 | 30 ± 25 | 34 ± 31 | 0.36 |
| Sandy loam till | 5 ± 8 | 4 ± 8 | 1 ± 2 | 0.62 |
| Fluvial Sand | 66 ± 19 | 48 ± 12 | 46 ± 28 | 0.24 |
| Organic | 11 ± 6 | 12 ± 7 | 14 ± 8 | 0.84 |
| Post-glacial sand | 3 ± 5 | 6 ± 8 | 4 ± 6 | 0.94 |
|  |  |  |  |  |
| Built up areas & roads | 7 ± 4 | 5 ± 2 | 5 ± 2 | 0.38 |
| Agriculture | 70 ± 15 | 75 ± 17 | 80 ± 6 | 0.45 |
| Forest and nature | 23 ± 16 | 20 ± 18 | 15 ± 7 | 0.68 |
