# Supplementary material for: Re-Meandering of Lowland Streams: Will Disobeying the Laws of Geomorphology Have Ecological Consequences?
Source: PLoS One. 2014 Sep 29;9(9):e108558. doi: 10.1371/journal.pone.0108558 (PMC4180926; doi:10.1371/journal.pone.0108558)
Supplement: Table S2 — In-stream substrate composition. Mean values are presented along with standard deviations (SD). Upper case letters indicate significant differences among stream types using one-way ANOVA and pair-wise Bonferroni corrected post hoc tests. (DOCX) [file pone.0108558.s003.docx]

|  | Stream type | | |
| --- | --- | --- | --- |
|  | Natural | Channelized | Restored |
| Cobble (>64 mm) | 3 ± 3^b^ | 1 ± 1^b^ | 19 ± 15^a^ |
| Pebble (16 – 64 mm) | 15 ± 14^ab^ | 7 ± 10^b^ | 30 ± 13^a^ |
| Gravel (2 – 16 mm) | 6 ± 3 | 7 ± 6 | 6 ± 4 |
| Sand (0.1 – 2 mm) | 66 ± 13^a^ | 71 ± 18^a^ | 34 ± 5^b^ |
| Clay | 11 ± 9 | 15 ± 3 | 11 ± 6 |
|  |  |  |  |
| Mud (FPOM) cover | 7 ± 2 | 26 ± 20 | 7 ± 6 |
| CPOM cover | 21 ± 4 | 20 ± 6 | 12 ± 9 |
