# Supplementary material for: Re-Meandering of Lowland Streams: Will Disobeying the Laws of Geomorphology Have Ecological Consequences?
Source: PLoS One. 2014 Sep 29;9(9):e108558. doi: 10.1371/journal.pone.0108558 (PMC4180926; doi:10.1371/journal.pone.0108558)
Supplement: Table S3 — Benthic macroinvertebrate taxa encountered across the 18 sites included in the survey. (DOCX) [file pone.0108558.s004.docx]

| Phylum/Class/Order | Family | Taxon (order/family/genus/species) |
| --- | --- | --- |
| Hydrozoa | Hydridae | *Hydra* sp. |
| Turbellaria | Dugesiidae | *Dugesia gonocephala* |
|  | Planariidae | *Polycelis nigra / tenuis* |
| Nematoda |  | Nematoda indet |
| Gastropoda | Ancylidae | *Acroloxus lacustris* |
|  |  | *Ancylus fluviatilis* |
|  | Hydrobiidae | *Bithynia tentaculata* |
|  |  | *Potamopyrgus antipodarum* |
|  | Lymnaeidae | *Lymnaea peregra* |
|  |  | *Lymnaea truncatula* |
|  | Physidae | *Physa fontinalis* |
|  | Planorbidae | *Gyraulus albus* |
|  |  | *Planorbis planorbis* |
| Lamellibranchia | Sphaeriidae | *Pisidium* sp. |
|  |  | *Sphaerium* sp. |
| Hirundinea | Erpobdellidae | *Erpobdella octoculata* |
|  | Glossiphoniidae | *Glossiphonia complanata* |
|  |  | *Glossiphonia concolor* |
|  |  | *Helobdella stagnalis* |
|  |  | *Theromyzon tessulatum* |
| Oligochaeta |  | Oligochaeta indet. |
| Malacostraca | Asellidae | *Asellus aquaticus* |
|  | Gammaridae | *Gammarus pulex* |
| Ephemeroptera | Baetidae | *Baetis niger* |
|  |  | *Baetis rhodani* |
|  |  | *Baetis vernus* |
|  |  | *Centroptilum luteolum* |
|  | Caenidae | *Caenis horaria* |
|  |  | *Caenis rivulorum* |
|  | Ephemerellidae | *Ephemerella ignita* |
|  | Ephemeridae | *Ephemera danica* |
|  | Heptagenidae | *Heptagenia fuscogrisea* |
|  |  | *Heptagenia sulphurea* |
|  | Leptophlebidae | *Leptophlebia marginata* |
|  |  | *Paraleptophlebia submarginata* |
|  | Siphlonuridae | *Metretopus borealis* |
| Odonata | Caleopteygidae | *Calopteryx splendens* |
|  |  | *Calopteryx virgo* |
|  | Coenagrionidae | *Pyrrhosoma nymphula* |

| *Table S5 continued* |  |  |
| --- | --- | --- |
| Phylum/Class/Order | Family | Taxon (order/family/genus/species) |
| Plecoptera | Leuctridae | *Leuctra fusca / digitata* |
|  |  | *Leuctra nigra* |
|  | Nemouridae | *Amphinemura standfussi / sulcicollis* |
|  |  | *Amphinemura sulcicollis* |
|  |  | *Nemoura avicularis* |
|  |  | *Nemoura cinerea* |
|  |  | *Nemoura flexuosa* |
|  |  | *Nemurella picteti* |
|  | Perlodidae | *Isoperla difformis* |
|  |  | *Isoperla grammatica* |
|  | Taeniopterygidae | *Brachyptera risi* |
| Heteroptera | Corixidae | *Paracorixa concinna* |
|  |  | *Sigara* sp. |
|  | Gerridae | *Gerris lacustris* |
|  | Nepidae | *Nepa cinerea* |
|  | Velidae | *Velia* sp. |
| Megaloptera | Sialidae | *Sialis fuliginosa* |
|  |  | *Sialis lutaria* |
| Coleoptera | Dryopidae | *Dryops* sp. |
|  | Dytiscidae | *Agabus* sp. |
|  |  | *Hydroporus* sp. |
|  |  | *Nebrioporus depressus* |
|  |  | *Oreodytes sanmarkii* |
|  |  | *Platambus maculatus* |
|  | Elmidae | *Elmis aenea* |
|  |  | *Limnius volckmari* |
|  |  | *Oulimnius tuberculatus* |
|  | Gyrinidae | *Gyrinus* sp. |
|  |  | *Orectochilus villosus* |
|  | Haliplidae | *Brychius elevatus* |
|  |  | *Haliplus* sp. |
|  | Helophoridae | *Helophorus* sp. |
|  | Hydraenidae | *Hydraena gracilis* |
|  | Hydrophilidae | *Anacaena limbata* |
|  |  | *Anacaena lutescens* |
|  |  | *Enochrus quadripunctatus / fuscipennis* |
|  | Scirtidae | *Elodes minuta* |

| *Table S5 continued* |  |  |
| --- | --- | --- |
| Phylum/Class/Order | Family | Taxon (order/family/genus/species) |
| Trichoptera | Beraeidae | *Beraeodes minutus* |
|  | Brachycentridae | *Brachycentrus maculatus* |
|  |  | *Brachycentrus subnubilus* |
|  | Goeridae | *Goera pilosa* |
|  |  | *Silo nigricornis* |
|  |  | *Silo pallipes* |
|  | Hydropsychidae | *Hydropsyche angustipennis* |
|  |  | *Hydropsyche pellucidula* |
|  | Hydroptilidae | *Hydroptila* sp. |
|  | Lepidostomatidae | *Lepidostoma hirtum* |
|  | Leptoceridae | *Adicella reducta* |
|  |  | *Athripsodes albifrons* |
|  |  | *Athripsodes albifrons / cinereus* |
|  |  | *Athripsodes aterrimus* |
|  |  | *Mystacides azurea* |
|  | Limnephilidae | *Anabolia nervosa* |
|  |  | *Chaetopteryx villosa* |
|  |  | *Ecclisopteryx dalecarlica* |
|  |  | *Glyphotaelius pellucidus* |
|  |  | *Halesus digitatus / tessulatus* |
|  |  | *Halesus radiatus* |
|  |  | *Limnephilus extricatus* |
|  |  | *Limnephilus lunatus* |
|  |  | *Limnephilus rhombicus* |
|  |  | *Potamophylax cingulatus* |
|  |  | *Potamophylax latipennis* |
|  |  | *Potamophylax latipennis / luctuosus* |
|  | Mollanidae | *Molanna angustata* |
|  | Polycentropodidae | *Cyrnus trimaculatus* |
|  |  | *Plectrocnemia conspersa* |
|  |  | *Polycentropus flavomaculatus* |
|  |  | *Polycentropus irroratus* |
|  | Psychomyidae | *Lype reducta* |
|  |  | *Psychomyia pusilla* |
|  | Rhyacophilidae | *Rhyacophila fasciata* |
|  |  | *Rhyacophila nubila* |
|  | Sericostomatidae | *Notidobia ciliaris* |
|  |  | *Sericostoma personatum* |
| Diptera | Athericidae | *Atherix ibis* |
|  | Ceratopogonidae | Ceratopogoninae indet |
|  | Chironomidae | Chironominae indet |
|  |  | Orthocladiinae indet |
|  |  | Prodiamesinae indet |
|  |  | Tanypodinae indet |
|  | Empididae | Empididae indet |
|  | Ephydridae | *Hydrellia* sp. |
|  | Limoniidae | Eriopterinae indet |
|  |  | Hexatominae indet |
|  | Pediciidae | *Dicranota* sp. |
|  | Psychodidae | Psychodinae indet |
|  | Ptychopteridae | *Ptychoptera* sp. |
|  | Simulidae | Simuliidae indet |
|  | Tabanidae | *Chrysops* sp. |
