# Supplementary material for: Re-Meandering of Lowland Streams: Will Disobeying the Laws of Geomorphology Have Ecological Consequences?
Source: PLoS One. 2014 Sep 29;9(9):e108558. doi: 10.1371/journal.pone.0108558 (PMC4180926; doi:10.1371/journal.pone.0108558)
Supplement: Table S4 — Benthic macroinvertebrates –10 common taxa in the different stream types. Mean abundance (per m2) is presented along with taxonomic names. (DOCX) [file pone.0108558.s005.docx]

|  | Stream type | | |
| --- | --- | --- | --- |
| Rank | Natural | Channelized | Restored |
| 1 | Oligochaeta indet  (2855) | Oligochaeta indet  (1873) | *Gammarus pulex*  (2444) |
| 2 | *Gammarus pulex*  (1364) | *Gammarus pulex*  (1607) | Oligochaeta indet  (1490) |
| 3 | Orthocladiinae indet  (1124) | Orthocladiinae indet  (871) | Orthocladiinae indet  (1345) |
| 4 | Chironominae indet  (468) | Chironominae indet  (667) | Chironominae indet  (926) |
| 5 | *Caenis rivulorum*  (424) | *Asellus aquaticus*  (173) | *Baetis rhodani*  (726) |
| 6 | Elmis aenea  (341) | *Pisidium* sp.  (170) | *Luctra fusca/digitata*  (327) |
| 7 | *Luctra fusca/digitata*  (285) | *Caenis rivulorum*  (150) | *Pisidium* sp.  (265) |
| 8 | *Baetis rhodani*  (274) | Tanypodinae indet  (106) | *Elmis aenea*  (258) |
| 9 | *Pisidium* sp.  (190) | *Luctra fusca/digitata*  (98) | *Caenis rivulorum*  (214) |
| 10 | Tanypodinae indet  (188) | Prodiamesinae indet  (76) | *Ancylus fluviatilis*  (103) |
