# Supplementary material for: Re-Meandering of Lowland Streams: Will Disobeying the Laws of Geomorphology Have Ecological Consequences?
Source: PLoS One. 2014 Sep 29;9(9):e108558. doi: 10.1371/journal.pone.0108558 (PMC4180926; doi:10.1371/journal.pone.0108558)
Supplement: Table S5 — River corridor land use characteristics of natural, channelized and restored streams. Mean values are presented along with standard deviations (SD). (DOCX) [file pone.0108558.s006.docx]

|  | Stream type | | |
| --- | --- | --- | --- |
|  | Natural  (n=6) | Channelized  (n=6) | Restored  (n=6) |
| Built-up areas & roads | 0 ± 0 | 0 ± 0 | 1 ± 2 |
| Agriculture | 10 ± 11 | 99 ± 2 | 70 ± 35 |
| Nature and wetlands | 90 ± 11 | 1 ± 2 | 29 ± 35 |
