# Supplementary material for: Re-Meandering of Lowland Streams: Will Disobeying the Laws of Geomorphology Have Ecological Consequences?
Source: PLoS One. 2014 Sep 29;9(9):e108558. doi: 10.1371/journal.pone.0108558 (PMC4180926; doi:10.1371/journal.pone.0108558)
Supplement: Table S6 — Water chemistry characteristics of the natural, channelized and restored streams. Mean values are presented along with standard deviations (SD). (DOCX) [file pone.0108558.s007.docx]

|  | Stream type | | |
| --- | --- | --- | --- |
|  | Natural | Channelized | Restored |
| pH | 7.54 ± 0.20 | 7.56 ± 0.50 | 7.43 ± 0.34 |
| Alkalinity (mM) | 1.93 ± 0.59 | 1.88 ±1.27 | 1.39 ± 1.31 |
| BOD_5_ (mg/L) | 1.3 ± 0.2 | 1.3 ± 0.3 | 1.2 ± 0.7 |
| Fe^2+^ (mg/L) | 0.05 ± 0.02 | 0.05 ± 0.01 | 0.04 ± 0.01 |
| NH_4_-N (µg/L) | 32.7 ± 10.8 | 33.2 ± 28.7 | 13.2 ± 9.0 |
| NO_3_-N (mg/L) | 2.48 ± 0.60 | 3.29 ± 2.16 | 2.68 ± 2.10 |
| Total N (mg/L) | 3.0 ± 0.8 | 3.68 ± 2.17 | 3.0 ± 2.1 |
| PO_4_-P (µg/L) | 10.3 ± 4.1 | 9.8 ± 3.5 | 9.8 ± 9.3 |
| Total P (µg/L) | 71.7 ± 36.5 | 66.3 ± 49.8 | 49.0 ± 50.9 |
