# Supplementary material for: Re-Meandering of Lowland Streams: Will Disobeying the Laws of Geomorphology Have Ecological Consequences?
Source: PLoS One. 2014 Sep 29;9(9):e108558. doi: 10.1371/journal.pone.0108558 (PMC4180926; doi:10.1371/journal.pone.0108558)
Supplement: Table S7 — Spearman rank correlation coefficients among the physico-chemical parameters from the stream reaches. P values are also presented in brackets (N = 18). Significance levels: *: 0.05; **: 0.01; ***:0.001 (DOCX) [file pone.0108558.s008.docx]

|  | Slope | Cobble | Pebble | Gravel | Sand | Clay | CPOM | Mud | Width_CV_ | Velocity_CV_ | Depth_CV_ | Sub Het | Width | Depth | BOD5 | pH | Alkalinity | Fe | NH_4_ | NO_3_ | TN | PO_4_ | TP |
| --- | --- | --- | --- | --- | --- | --- | --- | --- | --- | --- | --- | --- | --- | --- | --- | --- | --- | --- | --- | --- | --- | --- | --- |
| Catchment | 0,0661 | 0,6329** | 0,5282* | 0,1296 | -0,5356* | -0,0558 | -0,2171 | -0,1100 | 0,2962 | 0,0671 | 0,2900 | 0,1723 | 0,7317** | 0,5397* | 0,1847 | -0,0196 | 0,0558 | 0,4256 | 0,1221 | 0,3746 | 0,4241 | -0,1187 | 0,0279 |
|  | (0,7853) | (0,0091) | (0,0294) | (0,5931) | (0,0272) | (0,8180) | (0,3708) | (0,6503) | (0,2220) | (0,7821) | (0,2318) | (0,4773) | (0,0026) | (0,0261) | (0,4463) | (0,9356) | (0,8182) | (0,0793) | (0,6147) | (0,1225) | (0,0803) | (0,6246) | (0,9084) |
| Slope | - | 0,4757* | 0,6355** | -0,1432 | -0,3511 | -0,3071 | -0,3578 | -0,3122 | 0,5751* | 0,4522 | 0,4615 | 0,1105 | -0,0289 | -0,3304 | -0,0971 | -0,1094 | 0,0341 | -0,2586 | 0,0036 | -0,2571 | -0,2416 | 0,2523 | 0,0868 |
|  |  | (0,0498) | (0,0088) | (0,5550) | (0,1478) | (0,2055) | (0,1401) | (0,1980) | (0,0177) | (0,0622) | (0,0570) | (0,6487) | (0,9051) | (0,1731) | (0,6890) | (0,6518) | (0,8882) | (0,2863) | (0,9881) | (0,2891) | (0,3192) | (0,2981) | (0,7204) |
| Cobble |  | - | 0,6973** | -0,0182 | -0,7414** | -0,2040 | -0,5609* | -0,3130 | 0,3351 | 0,0628 | 0,4989* | 0,2776 | 0,5989* | 0,1191 | 0,0766 | 0,1447 | 0,2581 | 0,0038 | 0,2309 | 0,1159 | 0,1904 | 0,1152 | 0,1911 |
|  |  |  | (0,0040) | 0,9403 | (0,0022) | (0,4002) | (0,0207) | (0,1968) | (0,1671) | (0,7958) | (0,0397) | (0,2523) | (0,0135) | (0,6233) | (0,7522) | (0,5508) | (0,2873) | (0,9874) | (0,3411) | (0,6326) | (0,4324) | (0,6347) | (0,4306) |
| Pebble |  |  | - | 0,2679 | -0,7018** | -0,3132 | -0,4783* | -0,0682 | 0,5385* | 0,2532 | 0,3938 | 0,4992* | 0,3721 | -0,1292 | 0,3111 | 0,0258 | 0,2311 | 0,0037 | 0,4192 | 0,2760 | 0,3039 | 0,2425 | 0,3658 |
|  |  |  |  | 0,2693 | (0,0038) | (0,1966) | (0,0486) | (0,7786) | (0,0264) | (0,2964) | (0,1044) | (0,0396) | (0,1250) | (0,5942) | (0,1996) | (0,9151) | (0,3406) | (0,9878) | (0,0839) | (0,2552) | (0,2102) | (0,3173) | 0,1315) |
| Gravel |  |  |  | - | -0,1420 | 0,0862 | 0,1630 | 0,1599 | 0,1234 | -0,0435 | -0,0902 | 0,4676 | 0,2302 | -0,0747 | 0,1213 | 0,2768 | 0,3486 | 0,1402 | 0,2542 | 0,3888 | 0,3608 | 0,1464 | 0,4084 |
|  |  |  |  |  | (0,5581) | (0,7223) | (0,5015) | (0,5096) | (0,6109) | (0,8575) | (0,7100) | (0,0539) | (0,3426) | (0,7582) | (0,6170) | (0,2537) | (0,1507) | (0,5631) | (0,2947) | (0,1089) | (0,1368) | (0,5461) | 0,0922) |
| Sand |  |  |  |  | - | -0,0390 | 0,6264** | 0,1037 | -0,1373 | 0,2508 | -0,4654 | -0,7172** | -0,4469 | 0,0630 | -0,5397* | -0,3911 | -0,6288** | -0,0892 | -0,4056 | -0,3168 | -0,3932 | -0,3317 | -0,5393* |
|  |  |  |  |  |  | (0,8724) | (0,0098) | (0,6690) | (0,5714) | (0,3012) | (0,0550) | (0,0031) | (0,0654) | (0,7952) | (0,0261) | (0,1068) | (0,0095) | (0,7132) | (0,0945) | (0,1915) | (0,1050) | (0,1714) | (0,0262) |
| Clay |  |  |  |  |  | - | -0,0243 | 0,3526 | -0,1011 | -0,1737 | 0,0884 | 0,2758 | -0,1706 | -0,2253 | -0,0411 | 0,1095 | 0,2202 | 0,0038 | 0,0269 | 0,1527 | 0,1758 | 0,0551 | 0,1312 |
|  |  |  |  |  |  |  | (0,9203) | (0,1460) | (0,6769) | (0,4738) | (0,7154) | (0,2554) | (0,4819) | (0,3529) | (0,8656) | (0,6517) | (0,3640) | (0,9875) | (0,9116) | (0,5291) | (0,4685) | (0,8202) | (0,5885) |
| CPOM |  |  |  |  |  |  | - | 0,1447 | -0,0961 | 0,1127 | -0,1623 | -0,2977 | -0,1964 | 0,0599 | -0,1323 | -0,1809 | -0,2611 | 0,1403 | -0,1865 | -0,3070 | -0,3307 | -0,0939 | -0,3564 |
|  |  |  |  |  |  |  |  | (0,5506) | (0,6919) | (0,6423) | (0,5034) | (0,2197) | (0,4181) | (0,8048) | (0,5854) | (0,4558) | (0,2817) | (0,5629) | (0,4418) | (0,2056) | (0,1727) | (0,6985) | (0,1417) |
| Mud |  |  |  |  |  |  |  | - | 0,0492 | 0,0639 | -0,4514 | 0,3320 | -0,3508 | -0,1895 | 0,0806 | 0,0387 | 0,1132 | 0,1002 | -0,1585 | 0,1916 | 0,2063 | 0,1817 | 0,2306 |
|  |  |  |  |  |  |  |  |  | (0,8392) | (0,7922) | (0,0627) | (0,1711) | (0,1480) | (0,4345) | (0,7395) | (0,8731) | (0,6408) | (0,6796) | (0,5133) | (0,4294) | (0,3950) | (0,4537) | (0,3416) |
| Width_CV_ |  |  |  |  |  |  |  |  | - | 0,6904** | 0,2982 | 0,1765 | 0,0877 | -0,0753 | -0,1620 | -0,3746 | -0,2426 | -0,3864 | -0,1966 | -0,0464 | -0,0630 | -0,1208 | -0,1198 |
|  |  |  |  |  |  |  |  |  |  | (0,0044) | (0,2188) | (0,4669) | (0,7176) | (0,7561) | (0,5041) | (0,1225) | (0,3171) | (0,1112) | (0,4176) | (0,8481) | (0,7952) | (0,6184) | (0,6212) |
| Velocity_CV_ |  |  |  |  |  |  |  |  |  | - | 0,2301 | -0,2095 | -0,1166 | -0,0134 | -0,3664 | -0,5604* | -0,4729 | -0,2218 | -0,3859 | 0,0258 | 0,0010 | -0,3253 | -0,3192 |
|  |  |  |  |  |  |  |  |  |  |  | (0,3427) | (0,3877) | (0,6306) | (0,9559) | (0,1309) | (0,0209) | (0,0512) | (0,3604) | (0,1116) | (0,9153) | (0,9966) | (0,1798) | (0,1881) |
| Depth_CV_ |  |  |  |  |  |  |  |  |  |  | - | 0,2487 | 0,2487 | -0,1146 | 0,0155 | -0,3375 | -0,0785 | -0,0732 | 0,2100 | -0,1393 | -0,1001 | -0,2353 | -0,2789 |
|  |  |  |  |  |  |  |  |  |  |  |  | (0,3051) | (0,3051) | (0,6367) | (0,9491) | (0,1641) | (0,7463) | (0,7627) | (0,3865) | (0,5657) | (0,6798) | (0,3321) | (0,2501) |
| Sub Het |  |  |  |  |  |  |  |  |  |  |  | - | 0,1600 | -0,3127 | 0,5707* | 0,2982 | 0,5999* | -0,1369 | 0,4325 | 0,3849 | 0,4200 | 0,4663 | 0,6291** |
|  |  |  |  |  |  |  |  |  |  |  |  |  | (0,5096) | (0,1973) | (0,0186) | (0,2188) | (0,0134) | (0,5724) | (0,0746) | (0,1125) | (0,0833) | (0,0545) | (0,0095) |
| Width |  |  |  |  |  |  |  |  |  |  |  |  | - | 0,6698** | 0,0836 | 0,0774 | 0,0661 | 0,2845 | 0,0755 | 0,1496 | 0,1889 | -0,1590 | 0,1157 |
|  |  |  |  |  |  |  |  |  |  |  |  |  |  | (0,0058) | (0,7303) | (0,7496) | (0,7853) | (0,2408) | (0,7555) | (0,5372) | (0,4362) | (0,5122) | (0,6333) |
| Depth |  |  |  |  |  |  |  |  |  |  |  |  |  |  | -0,0753 | -0,1992 | -0,3211 | 0,5169* | -0,2897 | 0,1001 | 0,1022 | -0,5267* | -0,2665 |
|  |  |  |  |  |  |  |  |  |  |  |  |  |  |  | (0,7561) | (0,4115) | (0,1855) | (0,0331) | (0,2323) | (0,6798) | (0,6736) | (0,0299) | (0,2718) |
| BOD5 |  |  |  |  |  |  |  |  |  |  |  |  |  |  | - | 0,4675 | 0,6123* | 0,0170 | 0,4863* | 0,5129* | 0,5315* | 0,4334 | 0,5227* |
|  |  |  |  |  |  |  |  |  |  |  |  |  |  |  |  | (0,0539) | (0,0116) | (0,9442) | (0,0450) | (0,0345) | (0,0284) | (0,0739) | (0,0311) |
| pH |  |  |  |  |  |  |  |  |  |  |  |  |  |  |  | - | 0,9158*** | 0,0584 | 0,1500 | 0,2157 | 0,2570 | 0,6485** | 0,7758** |
|  |  |  |  |  |  |  |  |  |  |  |  |  |  |  |  |  | (0,0002) | (0,8098) | (0,5362) | (0,3738) | (0,2894) | (0,0075) | (0,0014) |
| Alkalinity |  |  |  |  |  |  |  |  |  |  |  |  |  |  |  |  | - | 0,0393 | 0,3002 | 0,3345 | 0,3913 | 0,7045** | 0,8481*** |
|  |  |  |  |  |  |  |  |  |  |  |  |  |  |  |  |  |  | (0,8713) | (0,2158) | (0,1678) | (0,1066) | (0,0037) | (0,0005) |
| Fe |  |  |  |  |  |  |  |  |  |  |  |  |  |  |  |  |  | - | 0,0702 | 0,0892 | 0,1199 | -0,2011 | 0,0106 |
|  |  |  |  |  |  |  |  |  |  |  |  |  |  |  |  |  |  |  | (0,7721) | (0,7132) | (0,6209) | (0,4070) | (0,9651) |
| NH_4_ |  |  |  |  |  |  |  |  |  |  |  |  |  |  |  |  |  |  | - | 0,3756 | 0,3735 | 0,4048 | 0,3635 |
|  |  |  |  |  |  |  |  |  |  |  |  |  |  |  |  |  |  |  |  | (0,1215) | (0,1236) | (0,0951) | (0,1339) |
| NO_3_ |  |  |  |  |  |  |  |  |  |  |  |  |  |  |  |  |  |  |  | - | 0,9917*** | 0,1409 | 0,4132 |
|  |  |  |  |  |  |  |  |  |  |  |  |  |  |  |  |  |  |  |  |  | (0,0000) | (0,5612) | (0,0884) |
| TN |  |  |  |  |  |  |  |  |  |  |  |  |  |  |  |  |  |  |  |  | - | 0,1738 | 0,4421 |
|  |  |  |  |  |  |  |  |  |  |  |  |  |  |  |  |  |  |  |  |  |  | (0,4736) | (0,0683) |
| PO_4_ |  |  |  |  |  |  |  |  |  |  |  |  |  |  |  |  |  |  |  |  |  | - | 0,7415** |
|  |  |  |  |  |  |  |  |  |  |  |  |  |  |  |  |  |  |  |  |  |  |  | (0,0022) |
